# Umbilical Hernia in Infants

**Published:** 1874-09

**Authors:** 


Umbilical Hernia in Infants.—A "Country Doctor" writes to the London *Medical Times and Gazette:*

Speediness in the cure of the above, combined with simplicity in the means employed, is, I hold, the great *desideratum.* What more simple than strips of plaster applied crosswise, or, as I have done during the last ten years, to apply a small pad of lint and one broad strip of adhesive plaster? No case has failed; no soreness have I ever seen, "so far as my memory serves me."

